# Pediatric diabetic retinopathy: experience of a tertiary hospital in Ethiopia

**DOI:** 10.1186/s13104-016-1941-6

**Published:** 2016-02-22

**Authors:** Mulugeta Sitot Shibeshi, Bereket Fantahun, Tedla Kebede, Birkneh Tilahun

**Affiliations:** Department of Pediatrics and Child Health, Hawassa University, Awassa, Ethiopia; Department of Pediatrics and Child Health, St. Paul Medical College, Addis Ababa, Ethiopia; Department of Internal Medicine, Addis Ababa University, Addis Ababa, Ethiopia

**Keywords:** Diabetes mellitus, Pediatrics, Retinopathy, Ethiopia

## Abstract

**Background:**

Diabetic retinopathy is one of the micro vascular complications of diabetes mellitus. To date there are no studies that show the magnitude of diabetic retinopathy in the pediatric population of Ethiopia with only very few in Africa. The purpose of this study was to determine the prevalence of diabetic retinopathy in children and adolescents at a tertiary center in Ethiopia.

**Methods:**

This cross-sectional hospital based descriptive study included children aged between 9 and 17 years attending the endocrine follow-up clinic of Tikur Anbesa Specialized Hospital. A structured questionnaire was used for evaluating sociodemographic data and information pertinent to diabetes. The prevalence of diabetic retinopathy was determined by fundus photography of each eye.

**Results:**

A total of 86 patients were examined with a mean age of 13.7 (SD = 1.8) years. At onset of diabetes, 95.6 % of children presented with diabetic ketoacidosis(DKA); 22 children (25.6 %) had at least two episodes of DKA, and 45 children (52.3 %) had poor glycemic control. Background retinopathy was present in four children (4.7 %) with a mean age of 14.25 (SD = 1.89) years and two of them also had maculopathy.

**Conclusion:**

Although there are some methodological limitations, this study highlights the difficulties of achieving good glycemic control and the early occurrence of diabetic retinopathy in Ethiopian diabetic children.

## Background

Diabetic retinopathy is one of the micro vascular complications of diabetes mellitus that results from damage to retinal capillaries and venules. It is the most common micro vascular disease seen in children and adolescents with type 1 diabetes and the most common cause of acquired blindness in young adults in United States [1, 2]. Prolonged duration of diabetes, poor glycemic control, hypertension, hyperlipidemia and genetic predisposition are risk factors for the development of diabetic retinopathy [3–6]. Family history of complications of diabetes and higher body mass index are other risk factors [7].

The results of prevalence studies of diabetic retinopathy are variable. A few studies have reported mild diabetic retinopathy in children with duration of disease as short as 1–2 years but in most studies the duration is 3 or more years, with typical durations of 8–10 years before development of retinopathy [8–11]. The prevalence of diabetic retinopathy ranged from 0–28 % in various studies [12–16]. Long-term metabolic control has a significant influence on the prevalence of retinopathy: in one study, it was found that patients with a median HbA1c ≥7.5 % had developed diabetic retinopathy on average after 15.5 years compared with 18.3 years in patients with lower HbA1c values [8].

There are several screening guidelines for pediatric diabetic retinopathy that are more or less similar recommending annual screening after 3–5 years of initial diagnosis of diabetes [17–19]. The International Society for Pediatric and Adolescent Diabetes (ISPAD) suggests that annual screening for retinopathy to be conducted in patients aged 11 years after diabetes of 2 years’ duration and from 9 years of age with diabetes of 5 years’ duration [4, 5].

To date there are no studies that show the magnitude of diabetic retinopathy in the pediatric population of Ethiopia with only very few in Africa. This has limited the attention to adopting any of the recognized screening tools to date. The current study assessed the prevalence and details of children with diabetic retinopathy strictly following the recommendations of ISPAD.

## Methods

### Design and setting

The cross-sectional descriptive study was conducted at Addis Ababa University, Collage of Health Sciencesand Pediatrics Endocrine Clinic in collaboration with the diabetic retinopathy screening program in the adult diabetes clinic of Tikur Anbessa Specialized Hospital that is found in the capital of Ethiopia, Addis Ababa.The pediatrics endocrine clinic is functional once weekly to evaluate patients on management and follow up. There are 212 type-1 diabetic patients having follow-up in the clinic, and on average 25–30 patients are evaluated on each follow-up day.

The ISPAD 2009 recommendation for retinopathy screening was followed to select the study subjects as it allows for the screening of children as young as 9 years with diabetic duration of 5 years or more [6]. All diabetic patients age 9 years and above with diabetic duration of 5 years and more, and diabetic patients whose age is greater than or equal to 11 years with diabetic duration of more than 2 years were selected. All of them were less than 18 years of age.

### Data collection

The selected patients who agreed to participate in the study were contacted by phone and were appointed for retinopathy screening. After receiving informed consent, the patients’ charts were reviewed, a standard questionnaire was administered and the patients, parents or guardians were interviewed and information pertinent to their diabetes (duration of diabetes, associated complications and co-morbidity) was collected.

Patients were asked if they had symptoms of neuropathy (numbness, pain and burning sensation of the hands and feet, and paresthesia) and the responses were recorded on the questionnaire. Patients, parents or guardians were asked if the study subjects had had screening laboratory tests for renal complication of diabetes (nephropathy). Those who had had the tests were asked if they had developed the renal complication. The responses were recorded on the questionnaire.

The vision of each eye was tested separately using Snellen distant vision chart at 6 m; uncorrected visual acuity of 6/6 or superior to 6/6 in both eyes was taken as normal [19].

Height and weight were measured without shoes. Body mass index (BMI) (weight in kg, divided by square of height in meters) was calculated, and BMI of <5th percentile andBMI of 95th percentile and above for age was taken as underweight and over-weight, respectively [20].

The results of HbA1c measurements of patients were documented, for those who had no HbA1c measurement, the average of the fasting blood sugar measurements of the last 1 month was taken to estimate the glycemic control. HbA1c results more than or equal to 10 %, and average fasting blood sugar level of more than 150 mg/dl for patients 11 years old and below, more than130 mg/dl for those between 12 years and 15 years of age, and more than 120 mg/dl for those between 16 and 18 years were taken as indicator of poor glycemic control [21].

Retinopathy was assessed using fundal photography of both eyes after dilating the pupils using 1 drop of tropicamide eye drops. The fundal photographs were taken with a Topcon Fundus Camera (TRC.NW 100; made in England) by nurses trained in digital diabetic retinopathy screening. Two fields of each eye (macular view and disc view) were photographed and the severity of retinopathy was determined by the grading of the most seriously affected eye. For the purpose of this study diabetic retinopathy was classified as follows [21]:

1. *Background or nonproliferative retinopathy:* cotton-wool spots, intra retinal hemorrhages, exudates, and micro vascular abnormalities (including micro aneurysms, occluded vessels, and dilated or tortuous vessels) primarily in the macula and posterior retina.
2. *Proliferative retinopathy*: presence of neovascularization arising from the disc and/or retinal vessels and the consequences of this neovascularization, including pre retinal and vitreous hemorrhage, subsequent fibrosis, and traction retinal detachment.
3. *Maculopathy*: The presence of exudates in the macula (region of the retina with-in a circular area of two disc diameters centered on the fovea) was considered as maculopathy as it is difficult to diagnose retinal thickening and edema from non-stereo color photographs.

### Statistical analysis

The data were entered in the Statistical Package for Social Sciences (SPSS inc.) software (version 20) data base. Data were presented as frequency (percentage), mean (standard deviation), and median (range). Chi square test or Fisher’s exact test was used to ascertain the association between categorical variables.Statistically significant association was taken for P values of <0.05.

### Ethical consideration

The study was conducted after obtaining ethical clearance from the Ethical committee of the Department of Pediatrics and Child Health, and the Institutional Review Board of Addis Ababa University, Collage of Health Sciences.

Written informed consent was obtained from the parents of participants for undergoing the study and for publication of the study results. Parental consent was also obtained to publish the clinical information contained in Table 2.

## Results

Among a total of 212 children who had follow up at Tikur Anbessa Specialized Hospital, 90 (45 %) of them full filled the criteria based on the recommendations of ISPAD and four of the selected didn’t appear for screening. The mean age of the participants was 13.7 (SD = 1.8) years with 95 % CI, 13.3–14.1. Females accounted for 54.7 %. The duration of follow up since the first diagnosis of diabetes ranged from 2–5 years in 35 (40.7 %); 6–10 years in 36 (41.9 %); and above 10 years in 15 (17.4 %) children. The age at diagnosis of diabetes was before 12 years for 95.3 % of the study subjects (Table 1). Most (83.7 %) of the diabetic children having follow-up at the diabetic clinic were from the city of Addis Ababa, and the remaining came from towns and villages surrounding the city.

**Table 1:** Clinical characteristics of the study subjects (n = 86)

Variables	Frequency	Percent
First presented with DKA		
Yes	83	96.5
No	3	3.5
Diabetic Duration		
2–5 years	35	40.7
6–10 years	36	41.9
> 10 years	15	17.4
Frequency of DKA since diagnosis		
1	64	74.4
2	17	19.8
3 and above	5	5.8
Diabetic nephropathy		
Yes	0	0
No	11	12.8
Never been screened	75	87.2
Neuropathy		
Yes	4	4.7
No	84	95.3
Previous retinopathy screening		
Yes	21	24.4
No	65	75.6
Body mass index		
Under weight	24	27.9
Normal	62	72.1
Over weight	0	0
Height for age		
Normal	69	80.5
Stunted	17	19.5
Hypertension		
Yes	1	1.16
No	85	98.84
Visual acuity		
Normal	68	79.1
Abnormal	18	20.9
Glycemic control		
Good	41	47.7
Poor	45	52.3
Retinopathy		
Yes	4	4.7
No	82	95.3
Stage of retinopathy		
Background/nonproliferative	4	100
Proliferative	0	0
Maculopathy	2	50

*DKA* Diabetic ketoacidosis

Among the 86 children included in the study, only 55(63.9 %) children had HbA_1c_measurements. Thirty nine (70.9 %) of them had two HbA1c measurements and the remaining 16 children had only one HbA1c throughout their follow up. Among the children who had HbA_1c_ measurement, 33 (60.0 %) had high (≥10 %) HbA_1c_ levels (average HbA_1C_ = 11.2 %). For the remaining 31 patients who did not have HbA_1c_ measurement, the average of the fasting blood sugar of the last 1 month from patient logs was taken as a measure of glycemic control. Eighteen (58.0 %) had high average 1 month fasting blood sugar levels (mean fasting blood glucose = 272.4 mg/dl, SD = 12.5). Overall, based on the few HbA_1c_ measurements and the average fasting blood sugar levels they had, it was apparent that 45 (52.3 %) patients had poor glycemic control that lasted for at least a few months.

The majority (95.6 %) of the study subjects presented with diabetic ketoacidosis (DKA) at onset of diabetes. Most children (74.4 %) had only one episode of DKA during their follow up; whereas 17 (19.8 %), 4 (4.7 %), and 1 (1.2) had twice, three and five times, respectively. Most of the study subjects measured their blood sugar only in the mornings except for occasional twice daily measurements but they did not test their urinary glucose.

Previous retinopathy screening during follow up had been done for only 21 (24.4 %) patients and two of the screened patients had had background retinopathy. Sixty eight (79.1 %) patients had normal visual acuity, while 18 (20.9 %) patients had abnormal visual acuity. Patients with abnormal visual acuity were referred to an ophthalmologist for evaluation. Only 11 patients (12.8 %) reported that they had had nephropathy screening and all had been told that they had no nephropathy. Four (4.7 %) patients had symptoms of neuropathy and they were linked to the pediatric neurology clinic for further evaluation.

Four of the 86 included children (4.7 %) fulfilled the diagnostic criteria for background diabetic retinopathy. Two of these patients also had maculopathy (background retinopathy involving the macula). The mean age of the children with diabetic retinopathy was 14.25 (SD = 1.89) years. The youngest child with retinopathy was 13 year old with diabetic duration of 10 years. No patient was found to have diabetic eye disease beyond background retinopathy in this study. All of them were female. The patients with maculopathy had reduced visual acuity but we did not know whether or not the maculopathy is responsible for their impaired vision. They were referred to an ophthalmologist for evaluation like the other patients with reduced visual acuity. All of the patients with retinopathy had high average HbA_1c_ level (mean HbA_1c_ = 12.4 %, SD = 0.88) and all presented with DKA at initial diagnosis. There was only one case with symptoms suggestive of peripheral neuropathy (Case 4; Table 2). All patients were taking a mixture of regular and Neutral Protamine Hagedorn (NPH) insulin subcutaneously (one third of the total daily dose being regular insulin and two third NPH). At the time of the study, two of the patients with retinopathy were taking 1 unit/kg insulin and the remaining two were taking 1.2 unit/kg insulin. Two third of the total dose was given in the morning and one third in the evening. Detailed description of the cases is presented in Table 2.

**Table 2:** Description of cases with diabetic retinopathy

Case	Age (years)	Sex	Age at Dx (years)	Average HbA_1c_ (%)	BMI	NEUR	NEP	V/A	Retinopathy stage	Maculopathy	DKAf	Insulin dose (u/kg)
1	14	F	2	12.5	N	None	No	6/6	Backgroud	No	1	1.2
2	13	F	3	11.2	N	None	UNK	6/12	Backgroud	Yes	3	1
3	17	F	6	13.3	N	None	UNK	6/6	Backgroud	No	2	1.2
4	13	F	3	12.7	N	Yes	UNK	6/12	Backgroud	Yes	2	1

*Yr* years, *Dx* diagnosis, *NEUR* neuropathy, *HbA1c* hemoglobin A1 C, *BMI* body mass index, *NEP* nephropathy, *DKAf* diabetic ketoacidosis frequency, *F* female, *N* normal, *V/A* visual acuity, *UNK* unknown, *U* unit, *kg* kilogram

## Discussion

The current study found that 90 (42.4 %) children having follow up at the diabetic clinic of Tikur Anbessa Specialized Hospital fulfilled the ISPAD criteria for undergoing diabetic retinopathy screening. All the screened patients were adolescents. The majority of the included children had a diabetic duration of 6–10 years.

As observed in this study our diabetic children did not have regular HbA_1c_ measurements making evaluation of long term metabolic control difficult. Among the study subjects, only 55 (63.9 %) had HbA_1c_measurements, the highest frequency being two measurements for their entire diabetic duration. Although we did not know their overall glycemic control throughout their diabetic duration, the few HbA_1c_ values and the 1 month average fasting blood glucose measurements reflected the clearly apparent poor glycemic control in more than half of the study subjects for at least a few months.

Despite the recommendations of ISPAD, little is known about the true impact of complications of diabetes, including diabetic retinopathy in the Ethiopian pediatric population. Screening for all of the microvarscular complications of diabetes was not routinely done indicating a suboptimal proactive follow up of these children. To our best knowledge this effort constitutes the first attempt to estimate the prevalence of diabetic retinopathy among pediatric diabetics in the country although the study subjects are not representative of the overall diabetic children in the country as they came from only the capital city and its surrounding areas.

The prevalence of pediatric diabetic retinopathy in pediatric diabetic patients having follow-up in Tikur Anbesa Specialized Hospital was found to be 4.7 %. Two patients also had associated maculopathy that could threaten their vision even though the stage of their retinopathy is background retinopathy. The reduced visual acuity in these two patients could be due to the maculopathy although further evaluation is needed to know the exact cause of their low visual acuity as it can also be affected by other factors like refractive error. The four patients with retinopathy and all those study subjects with reduced visual acuity were referred to an ophthalmologist for further evaluation, intervention and follow up.

Although we found out that all patients with retinopathy had high HbA_1c_ measurements, we could not conclude that these patients had long standing poor glycemic control as one or two HbA1c values will be insufficient to describe the long term metabolic control. It is well known that the development of retinopathy takes years and an HbA_1c_ measurement reflects the average blood glucose concentration from the preceding 2–3 months. Similarly, although it is well known from many other studies that prolonged poor glycemic control is associated with increased risk of development of retinopathy, we could not show this in our study due to the small number of our study subjects.

The finding that no patient was found to have proliferative retinopathy in our study is comparable to the findings elsewhere [22]. The prevalence of pediatric diabetic retinopathy in the current study is lower than that reported in other populations such as the study in Tanzania (22.2 %) [15], and Australia (8 % in children <11 years; 28 % in children >11 years) [16]. However, it is higher than those seen in other studies: France (4.5 %) [14] and Iceland (0 %) [12]. The substantial heterogeneity in reported prevalence of retinopathy may partly be real, for example due to genetic differences of different populations, but may also be due to differences in study methodology and population sample.The finding that there is poor glycemic control in the majority of our study subjects is similar to the findings in other African settings [15, 23].

The current study revealed that few patients had previous retinopathy screening despite the availability of the service through the Ethiopian Diabetic Association. The possible reasons for this, while they should be studied systematically, could be shortage of resources, low awareness of the practicing professionals in the center or failure to follow guidelines for follow up of such patients. This practice should be improved to detect the occurrence of diabetic retinopathy before patients develop vision threatening complication. Among the four patients with retinopathy, two had had retinopathy screening 1 year before the current evaluation that showed background retinopathy, and there was no progression of the stage of retinopathy after 1 year.

One of the major limitations of the current study is the inability to evaluate the long term glycemic control of the study subjects. Factors associated with the risk of developing diabetic retinopathy could not also be studied because of the small number of study subjects. The cause of the reduced visual acuity was not known in the patients with maculopathy as we could not get the ophthalmologist’s evaluation in time due to the long waiting list. Even though the center where the study was conducted is the largest national referral center for such cases, and many children from around the country come for follow up, the study still cannot be considered as representative of the pediatric diabetic population in the country. We also could not provide definition to the other complications of diabetes since we used chart review to obtain the data.

In conclusion, diabetic retinopathy, though uncommon, is a possible early complication in diabetic children in this setting. Hence, systematic screening should be in place for all micro vascular complications of diabetes.The results clearly show that major efforts are needed to improve quality of care in children with type 1 diabetes in Ethiopia. We recommend that children with diabetes should be followed for development of any complications using standard guidelines like ISPAD 2014. The patients with retinopathy should also be followed prospectively to evaluate the progression of their retinopathy. We also recommend more studies to assess the magnitude and the determinants of developing diabetic retinopathy, and the state of glycemic control and its determinants in the country using a large number of representative study subjects.
